# Association between weight change across adulthood and risk of chronic kidney disease: NHANES 1999–2020

**DOI:** 10.1080/0886022X.2024.2448261

**Published:** 2025-02-02

**Authors:** Xunliang Li, Mengqian Liu, Qihui Ye, Jiaxin Zhu, Wenman Zhao, Haifeng Pan, Deguang Wang

**Affiliations:** a Department of Nephrology, the Second Affiliated Hospital of Anhui Medical University, Hefei, China; b Institute of Kidney Disease, Inflammation & Immunity Mediated Diseases, The Second Hospital of Anhui Medical University, Anhui Medical University, Hefei, China; c Center for Big Data and Population Health of IHM, The Second Affiliated Hospital of Anhui Medical University, Hefei, China; d School of Nursing, Anhui Medical University, Hefei, China; e Department of Epidemiology and Biostatistics, School of Public Health, Anhui Medical University, Hefei, China

**Keywords:** Chronic kidney disease, weight change, obesity, risk factor, prevalence, NHANES

## Abstract

**Background:**

Obesity is a recognized risk factor for chronic kidney disease (CKD), but whether weight change is associated with CKD remains unclear. This research aimed to investigate the relationship between weight change patterns across adulthood and the risk of CKD.

**Methods:**

Data for 34,187 adults participating in the National Health and Nutrition Examination Survey 1999–2020 were analyzed. The weight change patterns of participants were assessed across different time intervals, including transitions from obesity to non-obesity, non-obesity to obesity, and remaining stable obesity. Absolute weight changes were also analyzed, categorizing participants into various weight gain and loss groups. Furthermore, stratified analyses were conducted to explore potential interactions between age, sex, and smoking status about CKD risk.

**Results:**

The study found that individuals transitioning from obesity to non-obesity, non-obesity to obesity, and remaining stable obesity had an elevated risk of developing CKD throughout adulthood compared to those maintaining stable non-obesity weight patterns. Moreover, a J-shaped or U-shaped relationship was observed between CKD risk and absolute weight changes, with both extreme weight gain (≥20 kg) and substantial weight loss (>2.5 kg) associated with increased CKD risk. Stratified analyses revealed that age and sex played significant roles in these associations, with stronger effects observed among participants under 60 years at baseline.

**Conclusions:**

This study underscores the link between weight change across adulthood and the risk of CKD. Maintaining a stable weight and avoiding extreme weight fluctuations may reduce CKD risk. These insights can be considered when developing CKD prevention and management strategies.

## Introduction

Chronic kidney disease (CKD) is a global health concern with a steadily increasing prevalence, placing a significant burden on healthcare systems worldwide [1]. This complex condition, characterized by a gradual decline in renal function over time, presents a substantial threat to public health due to its association with numerous complications, such as cardiovascular disease, hypertension, and metabolic disorders [2]. As research continues to explore the intricate web of factors contributing to CKD, growing attention has been focused on the relationship between changes in body weight throughout adulthood and the development of this condition [3]. Obesity, which involves the excessive accumulation of adipose tissue, has long been recognized as a risk factor for the development and progression of CKD [4]. However, many prior cohort studies have relied on a single body mass index (BMI) measurement, overlooking the dynamic nature of body weight over time [5]. According to the National Health and Nutrition Examination Survey (NHANES), adults gain weight more rapidly from young to middle adulthood, with excess adiposity mainly accruing during this period compared to the period from middle to late adulthood, when weight stabilizes or even decreases [6]. Additionally, preventing weight gain from young to middle adulthood might be more important than promoting weight loss because achieving long-term weight loss and maintaining it are challenging once a person develops obesity [7]. Therefore, further studies are needed to assess the impact of weight changes during specific life stages.

Although previous studies have reported that weight gain during adulthood is associated with an increased risk of CKD [8,9], the results have been inconsistent, with some studies showing no significant association [10]. Additionally, while some studies have found that weight loss is linked to a higher risk of CKD [3,11], others have reported the opposite [12,13]. Moreover, although the relationship between weight loss and CKD may depend to some extent on baseline BMI and the assessment of absolute weight change might underestimate the potentially detrimental effects of stable obesity, the effect of weight change patterns, considering the initial and change of obesity severity, on the CKD have never been comprehensively investigated in previous studies [14].

To address these critical knowledge gaps, we used updated data from NHANES 1999–2020 to examine the relationship between changes in weight from young adulthood to midlife and late adulthood with CKD.

## Methods

### Study design and participants

The NHANES is a program of studies designed and conducted by the National Center for Health Statistics. It aims to assess the health and nutritional status of the non-institutionalized population in the United States. The NHANES protocol was approved by the Institutional Review Board (IRB) of the National Center for Health Statistics, and the current study was exempt from ethical review by the IRB committee of our center because the research used publicly available deidentified secondary data.

This study was designed as a cross-sectional study using data from the NHANES 1999–2020, including participants aged 40 years or older. The exposures in this investigation focus on weight change patterns across adulthood. The outcome is CKD defined by estimated glomerular filtration rate (eGFR) and urinary albumin creatinine ratio (ACR) at baseline.

**Figure 1.**
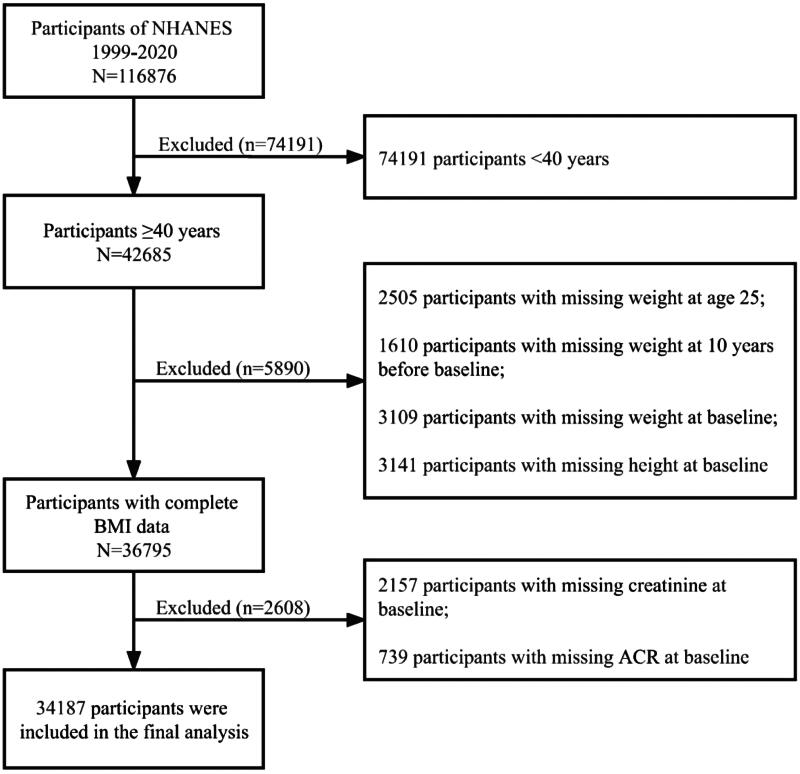
Participant selection. Abbreviations: ACR: albumin creatinine ratio; BMI: body mass index; CKD: chronic kidney disease; NHANES: national health and nutrition examination surveys.

Figure 1 shows a flow chart of participants’ selection. Of the 116,876 participants included in the NHANES 1999–2020 study, 74,191 participants younger than 40 were excluded. 2505 participants aged 25 years, 1610 participants at 10 years before baseline, and 3109 participants at baseline with missing recalled weight data were excluded. 3141 participants with missing baseline height measurements were further excluded. We also excluded 2157 participants with missing baseline creatinine measurements and 739 participants with missing baseline urinary ACR measurements. The final study cohort included 34,187 participants aged 40 years or older.

### Assessments of BMI and weight change pattern

Data on weight at age 25 and 10 years before the baseline NHANES survey were recalled at the baseline surveys. Baseline weight and height were measured during mobile physical examination. Body mass index (BMI) at age 25 years (BMI_25_), at 10 years before baseline survey (BMI_10prior_), and at baseline survey (BMI_baseline_) were calculated as recalled or survey-measured weight (kg) divided by the square of height (m^2^). Obesity was defined as BMI ≥30 kg/m^2^. We defined weight change patterns for three-time intervals: young to middle adulthood (BMI_25_ to BMI_10prior_), young to middle adulthood (BMI_10prior_ to BMI_baseline_), and whole adulthood period (BMI_25_ to BMI_baseline_). Four weight change patterns were identified for each interval: stable non-obesity (stay BMI < 30.0), obesity to non-obesity (BMI ≥30.0–<30.0), non-obesity to obesity (BMI < 30.0– ≥ 30.0), and stable obesity (stay BMI ≥30.0). The method has been described in detail in a previous publication using NHANES data [15]. Based on a recent study by Zheng et al. [16], we also classified absolute weight change in each time interval into five groups: weight change <2.5 kg (reference group), weight loss ≥2.5 kg, weight gain 2.5–9.9 kg, weight gain 10–19.9 kg, and weight gain ≥20.0 kg.

### The diagnosis of CKD

The KDIGO 2021 guideline was used to define CKD. The eGFR was calculated using the CKD Epidemiology Collaboration equation. CKD patients were defined if they were with eGFR <60 mL/min/1.73 m^2^ or urinary ACR ≥30 mg/g [17].

### Covariates measurements

Sociodemographic characteristics (age, sex, ethnicity, education level, marital status, family income-poverty ratio) were self-reported. Ethnicity was categorized into four groups (Non-Hispanic white, Non-Hispanic Black, Mexican American, and others). The family income-poverty ratio was calculated as total family income divided by the poverty threshold, categorized into three groups (0–1.0, 1.1–3.0, and >3.0). Education level was categorized into three groups (less than high school, high school or equivalent, and college or above), and marital status was categorized into three groups (married, separated, and never married). Current drinking and smoking status were self-reported.

### Statistical analysis

All data are presented as mean (*SD*) or number (percentage), as appropriate. Baseline characteristics were compared using the chi-square test for categorical variables and analysis of variance for continuous variables.

We first examined the relations between BMI at each time point and CKD. In this analysis, BMI was categorized into seven groups: underweight (<18.5), normal weight (18.5–24.9), overweight (25.0–29.9), and two categories of obesity (30.0–34.9 and ≥35.0), with 18.5–24.9 as the reference group. We examined the associations between the four weight change patterns in the three time intervals and CKD for the primary analyses. We also investigated the associations between absolute weight change groups in the above three time intervals and CKD and the possible non-linear relation by non-parametrically restricted cubic splines [18]. The above analyses used logistic regression models to determine odds ratios (ORs) and corresponding 95% confidence intervals (CIs). We made no adjustments in model 1. We adjusted for baseline age, sex, and ethnicity in model 2. We further adjusted for education level, marital status, family income-poverty ratio level, current drinking, and smoking status in model 3. We did stratified analyses and potential effect modifications by baseline age (<60 and ≥60 years), sex, and smoking status (ever and never smoking). A 2-tailed *p* < 0.05 was considered statistically significant in all analyses. Analyses were performed using R 4.2.2 software.

## Results

### Baseline characteristics according to weight change pattern

The study contained 34,187 participants in the final analysis. From age 25 years to baseline, 20,325 were stable non-obesity, 602 moved from obesity to non-obesity, 10,996 moved from non-obesity to obesity, and 2,264 were stable obesity (Table 1). From age, 25 years to 10 years before baseline, 24,087 of the participants were in the stable normal group, 365 of the participants were in the obesity to non-obesity group, 7,234 of the participants were in the non-obesity to obesity group, and 2,501 were in the stable obesity group (Supplementary Table 1). The corresponding numbers were 18,850; 2,077; 5,602 and 7,658 for the 10 years before baseline (Supplementary Table 2).

**Table 1.** Baseline characteristics of participants in NHANES 1999–2020 according to weight change patterns from age 25 years to baseline.

Characteristic	Total *N* = 34,187	Stable normal *N* = 20,325	Obese to non-obese *N* = 602	Non-obese to obese *N* = 10,996	Stable obese *N* = 2264	*p* Value
Age, years	57.3 ± 11.8	57.6 ± 12.2	58.8 ± 13.0	57.5 ± 11.2	53.5 ± 10.5	<0.001
Female	17,185 (52.1)	9,722 (51.7)	209 (37.1)	6,131 (54.8)	1,123 (45.8)	<0.001
Ethnicity						<0.001
Non-Hispanic white	16,023 (74.0)	9,928 (75.2)	263 (70.7)	4,857 (72.4)	975 (71.5)	
Non-Hispanic black	7,113 (9.5)	3,499 (7.6)	137 (12.4)	2,750 (11.8)	727 (14.9)	
Mexican American	5,033 (5.6)	2,761 (4.9)	126 (8.3)	1,832 (6.7)	314 (6.4)	
Others	6,018 (10.9)	4,137 (12.3)	76 (8.7)	1,557 (9.1)	248 (7.2)	
Education						<0.001
Less than high school	8,861 (16.0)	5,185 (15.7)	241 (26.9)	2,869 (16.4)	566 (15.6)	
High school or equivalent	8,042 (24.7)	4,701 (23.8)	124 (23.2)	2,661 (26.2)	556 (26.2)	
College or above	17,252 (59.3)	10,415 (60.6)	237 (49.9)	5,459 (57.4)	1,141 (58.1)	
Marital status						<0.001
Married	20,312 (65.1)	12,361 (66.2)	320 (57.3)	6,419 (64.2)	1,212 (60.9)	
Separated	10,275 (25.3)	5,902 (24.7)	209 (28.2)	3,451 (26.7)	713 (23.5)	
Never married	3,319 (9.6)	1,885 (9.0)	68 (14.4)	1,041 (9.1)	325 (15.6)	
Family income-poverty ratio level						<0.001
0–1.0	5,289 (10.4)	3,000 (9.8)	135 (17.2)	1,719 (10.6)	435 (13.3)	
1.1–3.0	13,009 (33.3)	7,581 (32.0)	263 (43.3)	4,285 (34.8)	880 (36.0)	
>3.0	12,831 (56.3)	7,921 (58.2)	145 (39.5)	4,018 (54.5)	747 (50.7)	
Current drinking	25,389 (82.3)	15,117 (83.1)	460 (85.1)	8,122 (81.2)	1,690 (79.9)	<0.001
Smoking status						<0.001
Never smoking	18,025 (53.1)	10,640 (53.0)	251 (39.5)	5,902 (53.3)	1,232 (55.9)	
Former smoking	9,976 (29.1)	5,661 (27.4)	172 (31.1)	3,504 (32.4)	639 (28.4)	
Current smoking	6,170 (17.8)	4,014 (19.6)	178 (29.5)	1,585 (14.3)	393 (15.8)	
BMI, kg/m^2^						
BMI_25_	23.9 ± 4.5	22.3 ± 2.8	33.5 ± 4.4	24.5 ± 3.0	34.7 ± 5.1	<0.001
BMI_10prior_	27.6 ± 6.1	24.7 ± 3.7	31.3 ± 5.8	30.7 ± 5.2	38.5 ± 7.7	<0.001
BMI_baseline_	29.2 ± 6.5	25.3 ± 3.0	26.8 ± 2.4	34.8 ± 4.5	39.6 ± 7.2	<0.001
eGFR, mL/min/1.73 m^2^	77.2 ± 31.7	77.9 ± 30.7	74.9 ± 34.9	75.8 ± 32.5	77.1 ± 35.7	<0.001
CKD	11,888 (26.8)	6,543 (24.4)	296 (36.8)	4,061 (29.7)	988 (33.2)	<0.001

Abbreviations: BMI: body mass index; CKD: chronic kidney disease; eGFR: estimated glomerular filtration rate; NHANES: national health and nutrition examination surveys.

According to the weight change patterns from age 25 years to baseline, compared with the other groups, those in the stable normal group tended to be non-Hispanic white, better educated, married, and richer; they were less likely to be former smoking (Table 1). Participants in the stable obesity group tended to be younger, whereas those in the obesity to non-obesity category tended to be older. Participants in the obesity to non-obesity group were more likely to be currently smoking, whereas the other two weight change groups were less likely to be currently smoking compared with the stable normal group. The distributions of participants’ characteristics according to the weight change pattern in the other two time periods are shown in Supplementary Tables 1 and 2, and the distribution patterns were generally similar except for some inconsistent patterns in age and current drinking.

### Relations of weight and weight change patterns with CKD

Among 34,187 participants, 11,888 CKD occurred. When evaluating the weight status at each time point, we found a linear association between BMI and CKD (Supplementary Table 3). For age 25 years, overweight and obesity were significantly associated with increased risks of CKD, whereas underweight was associated with decreased risks of CKD. The association between underweight and mortality became null as the participants aged.

Table 2 shows the association between weight change patterns in the three periods and CKD risk, using the stable normal group as the reference. Participants who transitioned from obesity to non-obesity, non-obesity to obesity, and stable obesity had an increased risk of CKD throughout adulthood compared to participants with stable non-obesity. For participants who transitioned from obesity to non-obesity, the ORs were 1.65 (95% CI 1.30–2.09) from age 25 to 10 years before baseline, 2.12 (95% CI 1.75–2.57) from age 25 to baseline, and 1.70 (95% CI 1.52–1.89) from 10 years before baseline. For participants who transitioned from non-obesity to obesity, the ORs were 1.48 (95% CI 1.39–1.57) from age 25 to 10 years before baseline, 1.37 (95% CI 1.29–1.45) from age 25 to baseline, and 1.28 (95% CI 1.19–1.38) from 10 years before baseline. For participants with stable obesity, the ORs were 2.20 (95% CI 2.00–2.43) from age 25 to 10 years before baseline, 2.19 (95% CI 1.98–2.43) from age 25 to baseline, and 1.72 (95% CI 1.61–1.83) from 10 years before baseline.

**Table 2.** Association between weight change patterns and CKD.

	Model 1			Model 2			Model 3		
Weight change patterns	OR	95% CI	*p* Value	OR	95% CI	*p* Value	OR	95% CI	*p* Value
From age 25 years to 10 years before baseline									
Stable non-obesity	1.00 (ref)	–	–	1.00 (ref)	–	–	1.00 (ref)	–	–
Obesity to non-obesity	1.61	1.30–1.98	<0.001	1.70	1.36–2.12	<0.001	1.65	1.30–2.09	<0.001
Non-obesity to obesity	1.63	1.54–1.72	<0.001	1.47	1.39–1.56	<0.001	1.48	1.39–1.57	<0.001
Stable obesity	1.81	1.67–1.97	<0.001	2.24	2.05–2.45	<0.001	2.20	2.00–2.43	<0.001
From age 25 years to baseline									
Stable non-obesity	1.00 (ref)	–	–	1.00 (ref)	–	–	1.00 (ref)	–	–
Obesity to non-obesity	2.04	1.73–2.40	<0.001	2.08	1.75–2.47	<0.001	2.12	1.75–2.57	<0.001
Non-obesity to obesity	1.23	1.17–1.29	<0.001	1.36	1.29–1.43	<0.001	1.37	1.29–1.45	<0.001
Stable obesity	1.63	1.49–1.78	<0.001	2.26	2.06–2.48	<0.001	2.19	1.98–2.43	<0.001
From 10 years before baseline to baseline									
Stable non-obesity	1.00 (ref)	–	–	1.00 (ref)	–	–	1.00 (ref)	–	–
Obesity to non-obesity	1.94	1.77–2.13	<0.001	1.71	1.55–1.88	<0.001	1.70	1.52–1.89	<0.001
Non-obesity to obesity	1.06	1.00–1.13	0.072	1.28	1.20–1.37	<0.001	1.28	1.19–1.38	<0.001
Stable obesity	1.62	1.53–1.71	<0.001	1.72	1.62–1.82	<0.001	1.72	1.61–1.83	<0.001

^a^No adjustments. ^b^Adjusted for baseline age, sex, and ethnicity. ^c^Adjusted for baseline age, sex, ethnicity, education level, marital status, family income-poverty ratio level, current drinker, and smoking status.

Abbreviations: CI: confidence interval; CKD: Chronic kidney disease; OR: odds ratio.

### Relations of absolute weight change with CKD

**Figure 2.**
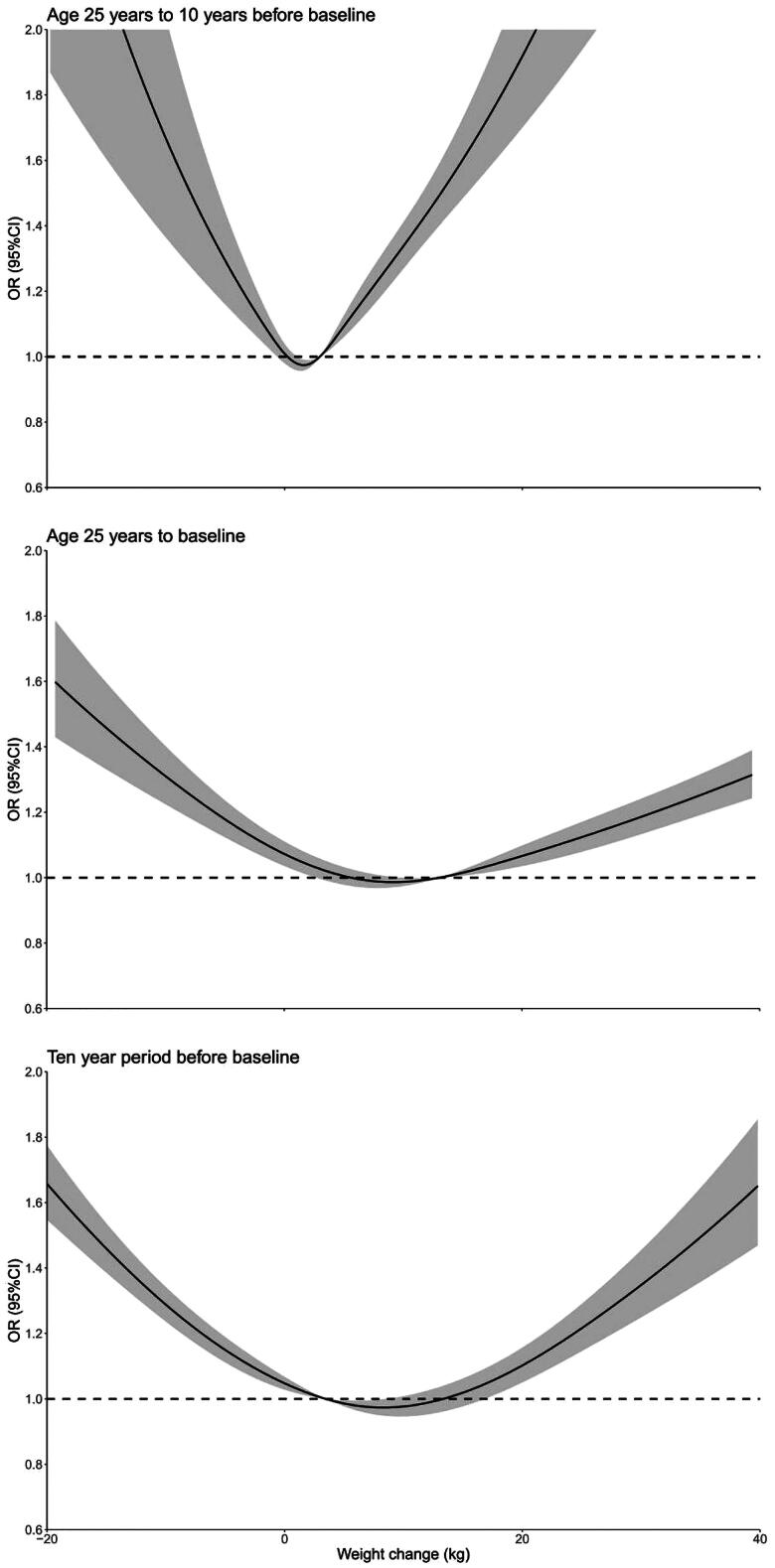
Dose-response association between absolute weight change across adulthood and CKD. Associations were examined by multivariable Logistic regression models based on restricted cubic splines. The solid line represents estimates of ORs, and the shaded part represents 95% CIs. Risk estimates were adjusted for baseline age, sex, ethnicity, education level, marital status, family income-poverty ratio level, current drinker, and smoking status. *p* Values for overall association and *p* values for non-linear association were all <0.001 in three periods. Abbreviations: CI: confidence interval; CKD: chronic kidney disease; OR: odds ratio.

When evaluating the absolute weight changes, we identified a J-shaped or U-shaped association between CKD and weight change across the three-time intervals (Figure 2). When classified (Table 3), the ORs for CKD in the extreme weight gain (weight gain ≥20 kg) group were 2.12 (95% CI 1.68–2.66) from age 25 years to 10 years before baseline, 1.20 (95% CI 1.08–1.32) from age 25 years to baseline, and 1.46 (95% CI 1.31–1.62) in the 10 years before baseline, compared with the stable weight group (weight change within 2.5 kg). Participants with moderate to large weight gain (weight gain ≥10 kg and <20 kg) from age 25 years to 10 years before baseline had an OR of 1.53 (95% CI 1.39–1.68) for CKD, with an OR of 1.46 (95% CI 1.31–1.62) in the age 25 years to baseline, whereas the association was not significant from 10 year period before baseline (OR 1.04, 95% CI 0.94–1.15). For participants with small to moderate weight gain (weight gain ≥2.5 kg and <10 kg), we observed a significant association with CKD only at age 25 years to 10 years before baseline (OR 1.14, 95% CI 1.08–1.20). Lost more than 2.5 kg were significantly associated with CKD in any of the three-time intervals (for age 25 years to 10 years before baseline, OR 1.37, 95% CI 1.17–1.59; for age 25 years to baseline, OR 1.35, 95% CI 1.20–1.51; for 10 year period before baseline, OR 1.49, 95% CI 1.39–1.61; respectively).

**Table 3.** Association between absolute weight change groups and CKD.

	Model 1^a^			Model 2^b^			Model 3^c^		
Absolute weight change groups	OR	95% CI	*p* Value	OR	95% CI	*p* Value	OR	95% CI	*p* Value
From age 25 years to 10 years before baseline									
Weight change within 2.5 kg	1.00 (ref)	–	–	1.00 (ref)	–	–	1.00 (ref)	–	–
Weight loss ≥2.5 kg	1.46	1.28–1.67	<0.001	1.40	1.21–1.61	<0.001	1.37	1.17–1.59	<0.001
Weight gain ≥2.5 kg and <10.0 kg	1.33	1.27–1.40	<0.001	1.13	1.07–1.19	<0.001	1.14	1.08–1.20	<0.001
Weight gain ≥10 kg and <20 kg	2.00	1.84–2.17	<0.001	1.56	1.43–1.70	<0.001	1.53	1.39–1.68	<0.001
Weight gain ≥20 kg	2.49	2.03–3.06	<0.001	2.18	1.76–2.70	<0.001	2.12	1.68–2.66	<0.001
From age 25 years to baseline									
Weight change within 2.5 kg	1.00 (ref)	–	–	1.00 (ref)	–	–	1.00 (ref)	–	–
Weight loss ≥2.5 kg	1.45	1.32–1.60	<0.001	1.37	1.24–1.52	<0.001	1.35	1.20–1.51	<0.001
Weight gain ≥2.5 kg and <10.0 kg	0.90	0.82–0.99	0.022	0.97	0.88–1.06	0.500	0.97	0.88–1.08	0.600
Weight gain ≥10 kg and <20 kg	0.98	0.90–1.07	0.600	1.07	0.97–1.17	0.200	1.04	0.94–1.15	0.400
Weight gain ≥20 kg	1.09	1.00–1.19	0.044	1.23	1.12–1.34	<0.001	1.20	1.08–1.32	<0.001
From 10 years before baseline to baseline									
Weight change within 2.5 kg	1.00 (ref)	–	–	1.00 (ref)	–	–	1.00 (ref)	–	–
Weight loss ≥2.5 kg	1.56	1.46–1.67	<0.001	1.49	1.39–1.60	<0.001	1.49	1.39–1.61	<0.001
Weight gain ≥2.5 kg and <10.0 kg	0.85	0.80–0.91	<0.001	1.01	0.94–1.08	0.800	0.98	0.91–1.06	0.600
Weight gain ≥10 kg and <20 kg	0.84	0.78–0.91	<0.001	1.16	1.07–1.26	<0.001	1.13	1.03–1.23	0.008
Weight gain ≥20 kg	1.00	0.91–1.09	0.900	1.50	1.37–1.66	<0.001	1.46	1.31–1.62	<0.001

^a^No adjustments. ^b^Adjusted for baseline age, sex, and ethnicity. ^c^Adjusted for baseline age, sex, ethnicity, education level, marital status, family income-poverty ratio level, current drinker, and smoking status.

Abbreviations: CI: confidence interval; CKD: Chronic kidney disease; OR: odds ratio.

### Stratified analyses

**Figure 3.**
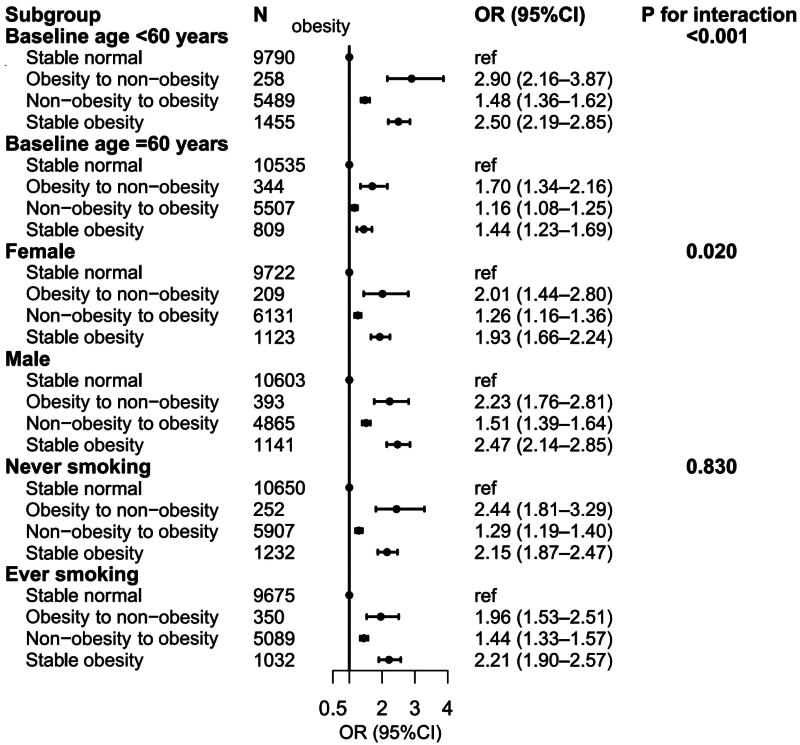
Associations between weight change patterns from age 25 years to baseline and CKD by stratified baseline age, sex, and smoking status in NHANES 1999–2020. Abbreviations: CKD: chronic kidney disease; NHANES: national health and nutrition examination surveys, OR: odds ratio.

In the stratified analyses from age 25 years to baseline, we found significant interactions with baseline age and sex but not with smoking status (Figure 3), and the associations were stronger among participants who were less than 60 years at baseline compared with their counterparts. We found similar results when we analyzed the age 25 years to 10 years before baseline and 10 years before baseline (Supplementary Figures 1 and 2).

## Discussion

Our findings demonstrated a clear association between BMI and CKD. At age 25, both overweight and obesity were associated with an increased risk of CKD, while underweight was linked to decreased risks. Interestingly, as participants aged, the association between underweight and CKD became null, underlining the dynamic nature of this relationship across the lifespan. Several factors could contribute to this dynamic relationship. The protective effect of underweight status observed at age 25 may be influenced by factors such as reduced metabolic demands and lower levels of inflammation, which may be more prevalent in younger individuals [19]. However, as participants age, other factors, such as the cumulative impact of aging, comorbid conditions, or changes in lifestyle and dietary habits, may attenuate this protective effect, resulting in the null association observed in later years [20]. This observation highlights the complexity of the relationship between underweight and CKD and suggests that the impact of underweight status on CKD risk is not static but evolves [21]. Further research is warranted to explore the underlying mechanisms and contributing factors that mediate this age-dependent association.

More notably, our study delved into the impact of weight change patterns throughout adulthood on CKD risk. Participants who transitioned from obesity to non-obesity, non-obesity to obesity, or remained stably obese experienced consistently elevated CKD risk compared to those who maintained stable non-obesity status. This finding aligns with previous research on the association between weight changes and CKD risk [22]. Several studies have reported that changes in body weight, particularly those involving transitions to or from obesity, can profoundly affect metabolic and renal function, ultimately contributing to an increased risk of CKD [21,23]. Our study found consistent results in this regard, further emphasizing the importance of considering weight change trajectories when assessing an individual’s risk for CKD [24]. The mechanisms underlying these associations are likely complex and multifaceted. Transitioning from obesity to non-obesity may involve metabolic changes, such as improved insulin sensitivity and reduced inflammation, which can positively affect kidney function [25]. On the other hand, transitioning from non-obesity to obesity may introduce metabolic disturbances detrimental to renal health. Staying stably obese is associated with a persistent burden of metabolic abnormalities, including hypertension and dyslipidemia, which can directly impact kidney function [26,27]. These findings underscore the significance of weight management throughout adulthood, particularly the avoidance of weight transitions involving obesity, as a potential strategy for reducing CKD risk.

When examining absolute weight changes, we observed a J-shaped or U-shaped association with CKD risk across three-time intervals, which aligns with previous research [27–29]. This pattern suggests that extreme weight gain and substantial weight loss can be associated with heightened CKD risk, emphasizing the importance of maintaining a stable weight within a healthy range. Weight gain, especially when substantial, can lead to metabolic disturbances, such as insulin resistance and inflammation, negatively impacting kidney function [30,31]. Conversely, rapid and significant weight loss can result in the loss of muscle mass, dehydration, and other factors that may adversely affect the kidneys [32,33]. Both excessive weight gain and rapid weight loss may have detrimental effects on renal health. When considering weight management strategies, healthcare providers and individuals should know these nuances in weight-related CKD risk and prioritize overall health and well-being [34].

We observed an intriguing pattern in our results: the observation that transitioning from obesity to non-obesity seems to carry at least as much, if not more, risk for CKD than moving in the opposite direction can be explained by several potential mechanisms. First, even gradual weight loss can lead to metabolic shifts that might not immediately benefit kidney function [35,36]. For example, weight loss may involve a reduction in lean muscle mass or changes in fat distribution, both of which could negatively affect renal health over time. Additionally, while transitioning from obesity to non-obesity might reduce inflammation and improve insulin sensitivity, the metabolic damage caused by years of obesity may persist, leading to sustained kidney damage [37]. Another factor to consider is weight cycling or ‘yo-yo dieting’, a common occurrence in individuals who lose significant weight only to regain it later [38]. This constant fluctuation can place stress on the kidneys and increase the risk of CKD [39,40]. Furthermore, unintentional weight loss, often a marker of underlying health issues such as chronic disease, could also contribute to higher CKD risk [41]. These complexities suggest that weight loss, even if not rapid, may still have detrimental long-term effects on kidney health, and further research is needed to explore the impact of weight fluctuations, including transitions from obesity to non-obesity, on kidney function.

Our stratified analyses revealed that the relationship between weight change patterns and CKD risk varied by age and sex but not by smoking status, which is consistent with previous findings. This underscores the importance of considering these demographic factors as potential effect modifiers when evaluating an individual’s risk for CKD. The greater prominence of associations among participants under 60 years at baseline is a significant and noteworthy finding. It suggests that younger individuals may be more susceptible to the effects of weight change patterns on CKD risk. This aligns with prior research indicating that younger age groups may be at higher risk for kidney disease in the context of obesity and weight fluctuations [21,33]. This observation emphasizes the importance of early intervention and prevention efforts, particularly among younger populations, to mitigate the impact of weight changes on CKD risk. In addition, the lack of significant interactions with smoking status is consistent with certain previous research [29,32]. This implies that, unlike age and sex, smoking status may not exert a substantial modifying effect on the relationship between weight changes and CKD risk. However, it is essential to acknowledge that smoking carries its own independent health risks, including potential effects on renal health, and should not be disregarded in the broader context of CKD prevention [42].

This study has several important limitations to consider when interpreting the findings. Firstly, weight gain and weight loss may have directly affected the renal disease markers used in the study (e.g., eGFR and urinary ACR). This effect of weight change on kidney function may have influenced, to some extent, the relationship we observed with CKD risk. Secondly, the analyses failed to control for key clinical confounders, such as hypertension and diabetes, particularly in the case of weight loss, which may be associated with underlying diseases that may themselves affect kidney function and thus may have an independent effect on CKD risk. Thirdly, recall bias from self-reported historical weight data and potential selection bias due to excluding participants with missing data may skew the sample representation. Fourthly, the cross-sectional design, while informative, lacks the temporal resolution of a time-dependent analysis, potentially obscuring the true relationship between weight changes and CKD risk over time. Fifthly, the analysis did not apply corrections for multiple comparisons, increasing the possibility of Type I errors (false positives). Sixthly, categorizing continuous variables like BMI, though partially mitigated by spline analysis for weight change, may result in a loss of nuanced information. Seventh, the lack of sensitivity analyses leaves the robustness of findings untested under varying assumptions. Lastly, using ORs for a common outcome like CKD may lead to overestimating relative risk, necessitating cautious interpretation. These limitations underscore the need for more comprehensive longitudinal studies to validate and refine the observed associations between weight change patterns and CKD risk.

## Conclusions

In conclusion, this study underscores the link between weight change across adulthood and the risk of CKD. Maintaining a stable weight and avoiding extreme weight fluctuations may reduce CKD risk. Clinicians and public health officials should consider these insights when developing CKD prevention and management strategies, particularly for younger populations.

## Supplementary Material

supplement.docx

## Data Availability

The National Health and Nutrition Examination Survey dataset is publicly available at the National Center for Health Statistics of the Center for Disease Control and Prevention (https://wwwn.cdc.gov/nchs/nhanes).
