# Supplementary material for: Association between weight change across adulthood and risk of chronic kidney disease: NHANES 1999–2020
Source: Ren Fail. 2025 Feb 2;47(1):2448261. doi: 10.1080/0886022X.2024.2448261 (PMC11792130; doi:10.1080/0886022X.2024.2448261)
Supplement: supplement.docx [file IRNF_A_2448261_SM1351.docx]

**Table S1. Baseline characteristics of participants in NHANES 1999-2020 according to weight change patterns from age 25 years to 10 years before baseline**

| **Characteristic** | **Total**  **N = 34187** | **Stable normal**  **N = 24087** | **Obese to non-obese**  **N = 365** | **Non-obese to obese**  **N = 7234** | **Stable obese**  **N = 2501** | ***P* value** |
| --- | --- | --- | --- | --- | --- | --- |
| Age, years | 57.3 ± 11.8 | 56.9 ± 11.8 | 56.5 ± 12.8 | 60.2 ± 11.5 | 54.1 ± 10.9 | <0.001 |
| Female | 17185 (52.1) | 12228 (53.7) | 153 (44.9) | 3625 (49.5) | 1179 (44.3) | <0.001 |
| Ethnicity |  |  |  |  |  | <0.001 |
| Non-Hispanic white | 16023 (74.0) | 11400 (74.0) | 190 (76.4) | 3385 (74.9) | 1048 (70.6) |  |
| Non-Hispanic black | 7113 (9.5) | 4567 (8.7) | 69 (9.3) | 1682 (10.5) | 795 (15.2) |  |
| Mexican American | 5033 (5.6) | 3383 (5.2) | 67 (7.7) | 1210 (6.5) | 373 (6.6) |  |
| Others | 6018 (10.9) | 4737 (12.0) | 39 (6.6) | 957 (8.0) | 285 (7.6) |  |
| Education |  |  |  |  |  | 0.009 |
| Less than high school | 8861 (16.0) | 6078 (15.8) | 138 (23.2) | 1976 (16.3) | 669 (16.8) |  |
| High school or equivalent | 8042 (24.7) | 5609 (24.3) | 77 (22.9) | 1753 (25.9) | 603 (26.1) |  |
| College or above | 17252 (59.3) | 12374 (60.0) | 150 (53.9) | 3500 (57.8) | 1228 (57.1) |  |
| Marital status |  |  |  |  |  | <0.001 |
| Married | 20312 (65.1) | 14571 (65.9) | 197 (57.8) | 4209 (64.2) | 1335 (60.6) |  |
| Separated | 10275 (25.3) | 6958 (24.8) | 131 (30.1) | 2395 (27.6) | 791 (23.5) |  |
| Never married | 3319 (9.6) | 2336 (9.3) | 34 (12.1) | 590 (8.2) | 359 (15.9) |  |
| Family income-poverty ratio level |  |  |  |  |  | <0.001 |
| 0-1.0 | 5289 (10.4) | 3618 (10.1) | 77 (14.6) | 1101 (10.0) | 493 (13.9) |  |
| 1.1-3.0 | 13009 (33.3) | 8917 (32.1) | 155 (41.0) | 2949 (36.3) | 988 (36.7) |  |
| >3.0 | 12831 (56.3) | 9427 (57.8) | 110 (44.4) | 2512 (53.7) | 782 (49.4) |  |
| Current drinking | 25389 (82.3) | 17889 (82.9) | 281 (82.0) | 5350 (80.9) | 1869 (80.6) | 0.015 |
| Smoking status |  |  |  |  |  | <0.001 |
| Never smoking | 18025 (53.1) | 12708 (53.0) | 149 (39.8) | 3834 (53.2) | 1334 (55.0) |  |
| Former smoking | 9976 (29.1) | 6726 (28.0) | 126 (32.6) | 2439 (33.3) | 685 (28.3) |  |
| Current smoking | 6170 (17.8) | 4640 (19.0) | 90 (27.5) | 959 (13.5) | 481 (16.7) |  |
| BMI, kg/m^2^ |  |  |  |  |  |  |
| BMI_25_ | 23.9 ± 4.5 | 22.4 ± 2.8 | 33.4 ± 4.2 | 25.2 ± 2.9 | 34.6 ± 5.1 | <0.001 |
| BMI_10prior_ | 27.6 ± 6.1 | 24.7 ± 3.0 | 27.1 ± 2.3 | 34.1 ± 4.4 | 38.8 ± 7.3 | <0.001 |
| BMI_baseline_ | 29.2 ± 6.5 | 26.9 ± 4.7 | 30.0 ± 6.2 | 34.3 ± 5.7 | 38.5 ± 7.9 | <0.001 |
| eGFR, mL/min/1.73 m^2^ | 77.2 ± 31.7 | 78.6 ± 30.6 | 77.6 ± 32.8 | 72.0 ± 33.6 | 76.6 ± 36.0 | <0.001 |
| CKD | 11888 (26.8) | 7529 (24.1) | 154 (32.1) | 3075 (34.0) | 1130 (34.0) | <0.001 |

Abbreviations: BMI, body mass index; CKD, chronic kidney disease; eGFR, estimated glomerular filtration rate; NHANES, national health and nutrition examination surveys,

**Table S2. Baseline characteristics of participants in NHANES 1999-2020 according to weight change patterns from 10 years before baseline to baseline**

| **Characteristic** | **Total**  **N = 34187** | **Stable normal**  **N = 18850** | **Obese to non-obese**  **N = 2077** | **Non-obese to obese**  **N = 5602** | **Stable obese**  **N = 7658** | ***P* value** |
| --- | --- | --- | --- | --- | --- | --- |
| Age, years | 57.3 ± 11.8 | 57.3 ± 12.1 | 61.3 ± 12.6 | 55.2 ± 10.8 | 58.0 ± 11.3 | <0.001 |
| Female | 17185 (52.1) | 9072 (52.1) | 859 (43.4) | 3309 (58.5) | 3945 (49.3) | <0.001 |
| Ethnicity |  |  |  |  |  | <0.001 |
| Non-Hispanic white | 16023 (74.0) | 9254 (75.2) | 937 (73.7) | 2336 (70.1) | 3496 (73.8) |  |
| Non-Hispanic black | 7113 (9.5) | 3199 (7.5) | 437 (10.1) | 1437 (12.7) | 2040 (12.1) |  |
| Mexican American | 5033 (5.6) | 2481 (4.7) | 406 (7.6) | 969 (7.1) | 1177 (6.3) |  |
| Others | 6018 (10.9) | 3916 (12.5) | 297 (8.6) | 860 (10.1) | 945 (7.8) |  |
| Education |  |  |  |  |  | <0.001 |
| Less than high school | 8861 (16.0) | 4736 (15.5) | 690 (20.8) | 1480 (17.3) | 1955 (15.4) |  |
| High school or equivalent | 8042 (24.7) | 4318 (23.5) | 507 (26.8) | 1368 (26.9) | 1849 (25.8) |  |
| College or above | 17252 (59.3) | 9775 (61.1) | 877 (52.4) | 2749 (55.8) | 3851 (58.8) |  |
| Marital status |  |  |  |  |  | 0.002 |
| Married | 20312 (65.1) | 11538 (66.4) | 1143 (61.5) | 3230 (63.6) | 4401 (63.7) |  |
| Separated | 10275 (25.3) | 5391 (24.5) | 720 (28.1) | 1698 (26.0) | 2466 (26.2) |  |
| Never married | 3319 (9.6) | 1749 (9.1) | 204 (10.4) | 621 (10.3) | 745 (10.1) |  |
| Family income-poverty ratio level |  |  |  |  |  | <0.001 |
| 0-1.0 | 5289 (10.4) | 2755 (9.7) | 380 (13.0) | 940 (11.8) | 1214 (10.5) |  |
| 1.1-3.0 | 13009 (33.3) | 6956 (31.6) | 888 (39.8) | 2116 (34.2) | 3049 (35.6) |  |
| >3.0 | 12831 (56.3) | 7485 (58.7) | 581 (47.3) | 2052 (53.9) | 2713 (53.9) |  |
| Current drinking | 25389 (82.3) | 14035 (83.3) | 1542 (81.5) | 4135 (81.4) | 5677 (80.7) | <0.001 |
| Smoking status |  |  |  |  |  | <0.001 |
| Never smoking | 18025 (53.1) | 9899 (53.0) | 992 (48.7) | 2958 (52.3) | 4176 (54.8) |  |
| Former smoking | 9976 (29.1) | 5188 (27.3) | 645 (30.0) | 1664 (30.7) | 2479 (32.5) |  |
| Current smoking | 6170 (17.8) | 3753 (19.7) | 439 (21.3) | 977 (17.0) | 1001 (12.7) |  |
| BMI, kg/m^2^ |  |  |  |  |  |  |
| BMI_25_ | 23.9 ± 4.5 | 22.2 ± 2.9 | 25.9 ± 4.7 | 23.8 ± 3.3 | 28.1 ± 5.5 | <0.001 |
| BMI_10prior_ | 27.6 ± 6.1 | 24.1 ± 2.9 | 33.2 ± 4.2 | 27.0 ± 2.3 | 35.8 ± 5.9 | <0.001 |
| BMI_baseline_ | 29.2 ± 6.5 | 25.1 ± 3.0 | 27.5 ± 2.1 | 33.6 ± 3.6 | 37.2 ± 5.9 | <0.001 |
| eGFR, mL/min/1.73 m^2^ | 77.2 ± 31.7 | 78.4 ± 30.4 | 71.3 ± 34.4 | 79.2 ± 31.2 | 73.6 ± 34.2 | <0.001 |
| CKD | 11888 (26.8) | 5868 (23.6) | 971 (36.9) | 1815 (26.3) | 3234 (33.4) | <0.001 |

Abbreviations: BMI, body mass index; CKD, chronic kidney disease; eGFR, estimated glomerular filtration rate; NHANES, national health and nutrition examination surveys.

**Table S3. Association between BMI and CKD at different time points NHANES 1999-2020**

|  | **Model 1^a^** | | | **Model 2^b^** | | | **Model 3^c^** | | |
| --- | --- | --- | --- | --- | --- | --- | --- | --- | --- |
| **BMI groups** | **OR** | **95% CI** | ***P* value** | **OR** | **95% CI** | ***P* value** | **OR** | **95% CI** | ***P* value** |
| BMI at age 25 years | | | | | | | | | |
| <18.5 kg/m^2^ | 0.82 | 0.74-0.91 | <0.001 | 0.90 | 0.80-1.01 | 0.070 | 0.88 | 0.77-0.99 | 0.038 |
| 18.5-24.9 kg/m^2^ | 1.00 (ref) | - | - | 1.00 (ref) | - | - | 1.00 (ref) | - | - |
| 25.0-29.9 kg/m^2^ | 1.22 | 1.15-1.28 | <0.001 | 1.32 | 1.25-1.40 | <0.001 | 1.36 | 1.28-1.45 | <0.001 |
| 30.0-34.9 kg/m^2^ | 1.60 | 1.46-1.76 | <0.001 | 1.97 | 1.78-2.18 | <0.001 | 1.95 | 1.75-2.17 | <0.001 |
| ≥35.0 kg/m^2^ | 1.78 | 1.56-2.04 | <0.001 | 2.52 | 2.19-2.90 | <0.001 | 2.47 | 2.11-2.89 | <0.001 |
| BMI at ten years before baseline | | | | | | | | | |
| <18.5 kg/m^2^ | 0.89 | 0.72-1.10 | 0.300 | 1.21 | 0.97-1.50 | 0.089 | 1.24 | 0.97-1.58 | 0.082 |
| 18.5-24.9 kg/m^2^ | 1.00 (ref) | - | - | 1.00 (ref) | - | - | 1.00 (ref) | - | - |
| 25.0-29.9 kg/m^2^ | 1.40 | 1.33-1.48 | <0.001 | 1.25 | 1.18-1.33 | <0.001 | 1.26 | 1.18-1.34 | <0.001 |
| 30.0-34.9 kg/m^2^ | 1.81 | 1.69-1.93 | <0.001 | 1.62 | 1.51-1.74 | <0.001 | 1.64 | 1.52-1.77 | <0.001 |
| ≥35.0 kg/m^2^ | 2.32 | 2.14-2.50 | <0.001 | 2.26 | 2.09-2.45 | <0.001 | 2.24 | 2.05-2.45 | <0.001 |
| BMI at baseline | | | | | | | | | |
| <18.5 kg/m^2^ | 1.20 | 0.97-1.47 | 0.094 | 1.20 | 0.96-1.50 | 0.100 | 1.20 | 0.93-1.54 | 0.200 |
| 18.5-24.9 kg/m^2^ | 1.00 (ref) | - | - | 1.00 (ref) | - | - | 1.00 (ref) | - | - |
| 25.0-29.9 kg/m^2^ | 1.04 | 0.98-1.11 | 0.200 | 1.08 | 1.01-1.15 | 0.020 | 1.08 | 1.01-1.16 | 0.030 |
| 30.0-34.9 kg/m^2^ | 1.21 | 1.13-1.29 | <0.001 | 1.34 | 1.25-1.43 | <0.001 | 1.34 | 1.25-1.45 | <0.001 |
| ≥35.0 kg/m^2^ | 1.44 | 1.34-1.54 | <0.001 | 1.81 | 1.68-1.95 | <0.001 | 1.80 | 1.65-1.95 | <0.001 |

^a^No adjustments. ^b^Adjusted for baseline age, sex, and ethnicity. ^c^Adjusted for baseline age, sex, ethnicity, education level, marital status, family income-poverty ratio level, current drinker, and smoking status.

Abbreviations: BMI, body mass index; CI, confidence interval; CKD, chronic kidney disease; NHANES, national health and nutrition examination surveys, OR, odds ratio.


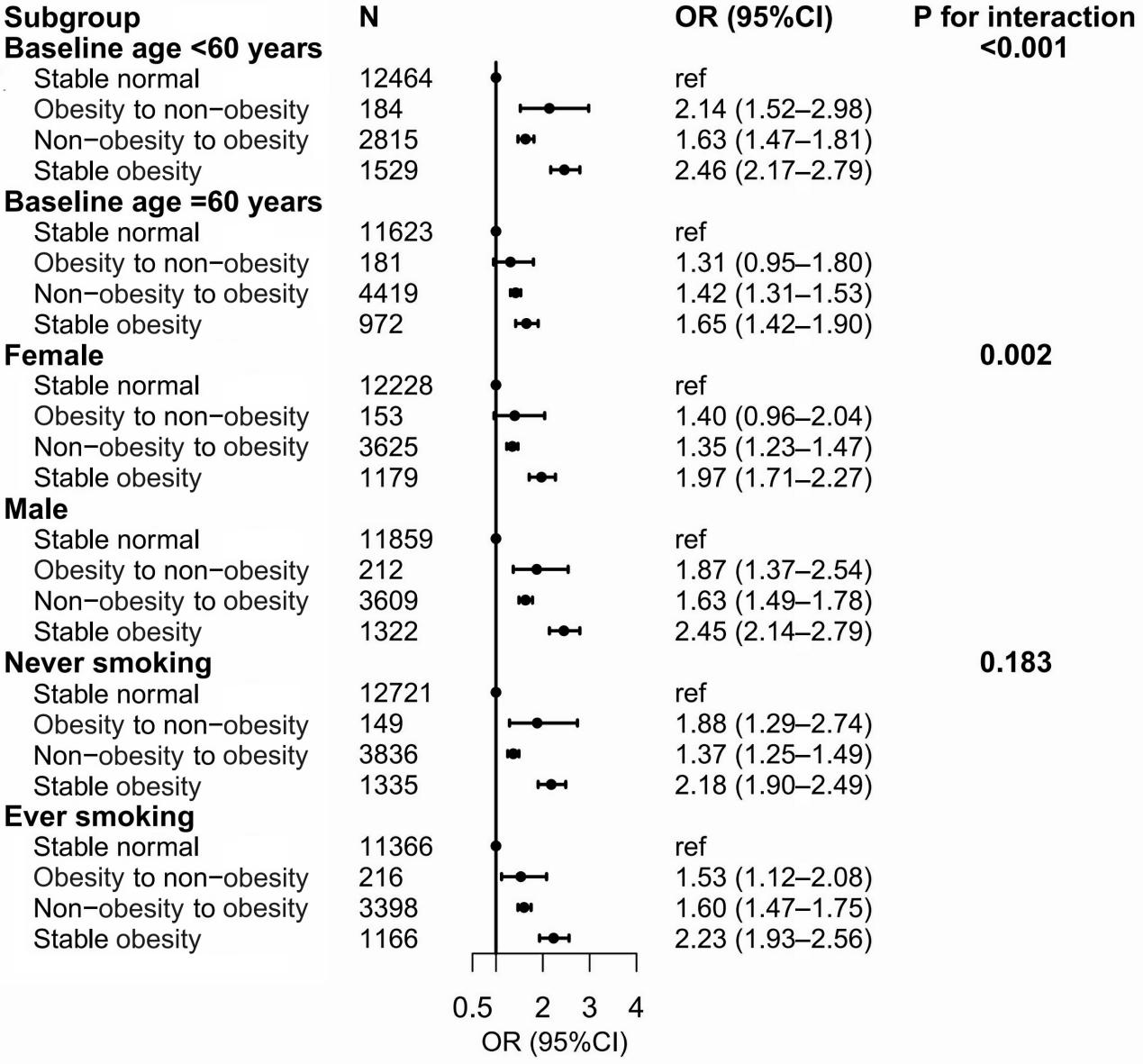


**Figure S1. Associations** **between weight change patterns from age 25 years to 10 years before baseline and CKD by stratified baseline age, sex, and smoking status in NHANES 1999-2020**

Abbreviations: CKD, chronic kidney disease; NHANES, national health and nutrition examination surveys, OR, odds ratio.


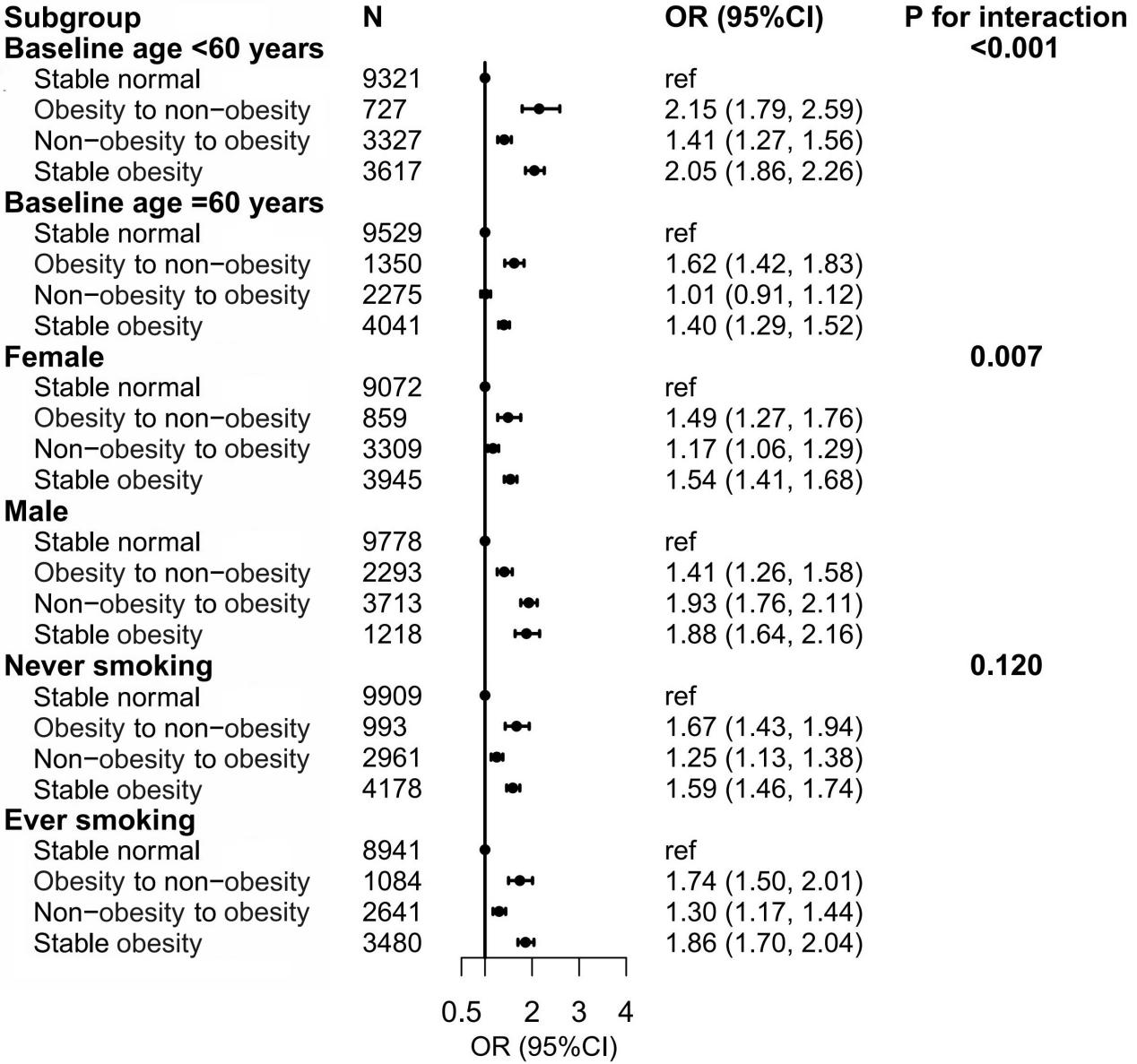


**Figure S2. Associations between weight change patterns from 10 years before baseline to baseline and CKD by stratified baseline age, sex, and smoking status in NHANES 1999-2020**

Abbreviations: CKD, chronic kidney disease; NHANES, national health and nutrition examination surveys, OR, odds ratio.
